# Carotid Cavernous Fistula: Ophthalmological Implications

**DOI:** 10.4103/0974-9233.53862

**Published:** 2009

**Authors:** Imtiaz A. Chaudhry, Sahar M. Elkhamry, Waleed Al-Rashed, Thomas M. Bosley

**Affiliations:** From the Oculoplastic and Orbit Division, King Khaled Eye Specialist Hospital, Riyadh, Saudi Arabia; 1 From the Radiology Department, King Khaled Eye Specialist Hospital, Riyadh, Saudi Arabia; 2 From the Anterior Segment Division, King Khaled Eye Specialist Hospital, Riyadh, Saudi Arabia; 3 From the Department of Ophthalmology, King Saud University, Riyadh, Saudi Arabia

**Keywords:** Carotid, Cavernous Sinus, Diagnosis, Fistula, Ophthalmological Findings, Treatment

## Abstract

Carotid cavernous fistula (CCF) is an abnormal communication between the cavernous sinus and the carotid arterial system. A CCF can be due to a direct connection between the cavernous segment of the internal carotid artery and the cavernous sinus, or a communication between the cavernous sinus, and one or more meningeal branches of the internal carotid artery, external carotid artery or both. These fistulas may be divided into spontaneous or traumatic in relation to cause and direct or dural in relation to angiographic findings. The dural fistulas usually have low rates of arterial blood flow and may be difficult to diagnose without angiography. Patients with CCF may initially present to an ophthalmologist with decreased vision, conjunctival chemosis, external ophthalmoplegia and proptosis. Patients with CCF may have predisposing causes, which need to be elicited. Radiological features may be helpful in confirming the diagnosis and determining possible intervention. Patients with any associated visual impairment or ocular conditions, such as glaucoma, need to be identified and treated. Based on patient's signs and symptoms, timely intervention is mandatory to prevent morbidity or mortality. The conventional treatments include carotid ligation and embolization, with minimal significant morbidity or mortality. Ophthalmologist may be the first physician to encounter a patient with clinical manifestations of CCF, and this review article should help in understanding the clinical features of CCF, current diagnostic approach, usefulness of the available imaging modalities, possible modes of treatment and expected outcome.

## INTRODUCTION

**Figure 1:**
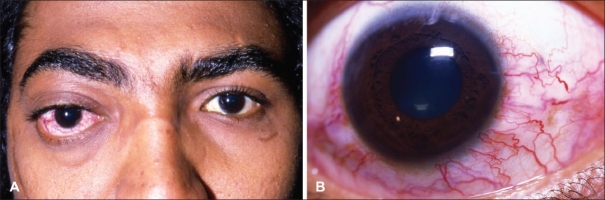
This 25-year-old man presented with chief complaints of right eye proptosis, decreased vision and elevated intraocular pressure (A). Closer examination revealed dilated episcleral vessels. Based on history and imaging studies, diagnosis of CCF was made (B)

Carotid cavernous fistula (CCF) results from abnormal communication between previously normal carotid artery and cavernous sinus. The most common (70%-90%) etiology of direct CCF is trauma from a basal skull fracture resulting in tear in the internal carotid artery (ICA) within the cavernous sinus.^1^–^3^ Motor vehicle accidents, falls and other crush injuries contribute to the incidence of basilar skull fractures and the formation of some of the CCFs. These patients may present with signs and symptoms such as conjunctival chemosis, proptosis, pulsating exophthalmos, diplopia, ophthalmoplegia, orbital pain, audible bruits and blindness.^2^ Causes that are not very common include spontaneous rupture of an existing aneurysm or atherosclerotic artery, usually in postmenopausal, hypertensive females.^1^–^3^ Small meningeal arteries supplying dural wall of cavernous sinus can rupture spontaneously while ICA itself may remain intact. These fistulas usually result in less severe symptoms, with insidious onset, mild orbital congestion, proptosis and low or no bruit.^1^–^6^ Patients may present with limbal injection, arterialized conjunctival and episcleral vessels (Figure 1). Fistulas may fluctuate or resolve spontaneously. Patients with CCF initially may present to an ophthalmologist for their eye symptoms.^4^ Careful history, examination and, in many instances, diagnostic imaging may lead to correct and timely diagnosis. Most studies of patients with CCF having ophthalmological complaints have come from the western countries,^7^–^10^ and studies of such patients have rarely been reported from the Middle East and African countries. The purpose of this review article is to describe demographic and clinical features, including ophthalmological complaints, radiological features and current treatment strategies employed, of suspected and proven cases of CCFs in patients presenting to an ophthalmologist.

## OPHTHALMOLOGICAL FEATURES

**Figure 2:**
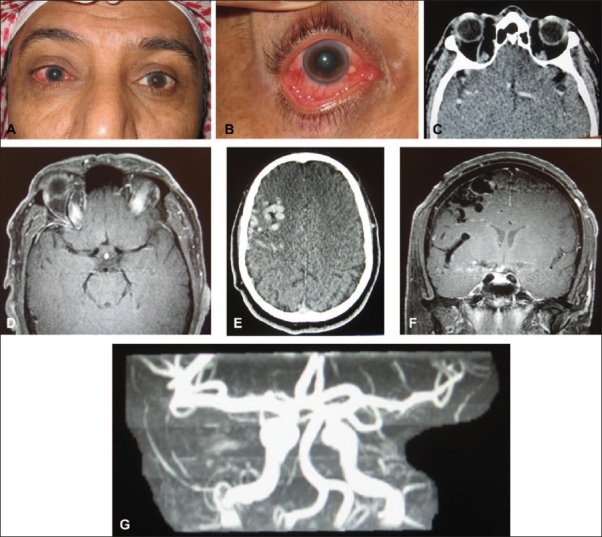
This 51-year-old man presented with right-sided proptosis, dilated pupil, elevated intraocular pressure and dilated episcleral vessels (A, B). CT scan and MRI revealed enlarged right-sided SOV (C, D) associated with cerebral signal void (E, F), suggestive of CCF. MRA confirmed presence of CCF (G)

**Figure 3:**
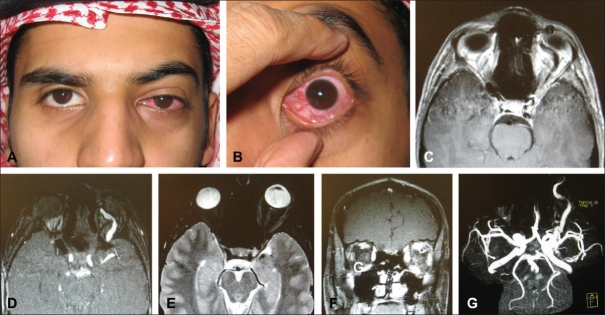
This 17-year-old male presented with sudden development of decreased vision in the left eye, proptosis, conjunctival chemosis, ptosis and elevated intraocular pressure 1 year after having experienced head trauma (A, B). Imaging studies revealed classic features of CCF on the left side manifested as evidence of the enlargement of the superior ophthalmic vein (SPV)(C). Dilated signal-void serpiginous structures are seen intraconally and extending to the left cavernous sinus (C, D). In addition, there were enlarged extraocular muscles on the left side, as evidenced by axial and cornonal MRI (E, F). MRA and MRV confirmed the diagnosis of CCF with markedly enlarged left SOV (G)

Patients with CCF may present with pulsatile exophthalmos, chemosis and complaints of hearing a noise in the head.^1^–^13^ Patients who present with corkscrew episcleral blood vessels in association with conjunctival chemosis, pulsating proptosis, thrill and bruit should raise the possibility of a diagnosis of arteriovenous fistula (Figures 1–3).^12^ Pathophysiology of proptosis, episcleral and conjunctival arterializations may be due to the resistance from the retrograde venous drainage into the ophthalmic vein. One may expect restricted ocular motility and diplopia as a result of enlargement of extraocular muscles; and exposure keratopathy as a result of proptosis (Figures 3E and 3F). These patients may present with a painful cranial nerve palsy with a white quiet eye in the absence of any proptosis.^14^ Careful auscultation may reveal an orbital bruit, and magnetic resonance imaging (MRI) may demonstrate abnormal dural-based enhancement. The definite diagnosis can be confirmed by angiography, which may confirm a posterior draining CCF (Figure 3G). Chemosis and episcleral congestion may present with red eye.^4^ Increased episcleral pressure and vortex venous pressure may result in elevated intraocular pressure (IOP) and secondary glaucoma.^15^–^17^ Secondary glaucoma is a frequently observed ocular manifestation of CCF, and closure of the fistula is the primary condition required for favorable IOP control. Talks *et al*.^15^ reported a case of angle-closure glaucoma secondary to the rapid development of a choroidal effusion in a patient with a longstanding CCF. It was thought that the development of the choroidal effusion occurred because of partial thrombosis of the ipsilateral superior ophthalmic vein (SOV) and cavernous sinus. Drainage of the choroidal effusion resolved the angle-closure glaucoma. Although glaucoma is usually associated with increased episcleral venous pressure, yet it may be due to iris neovascularization as a result of retinal ischemia.^18^ Ishijima *et al*.^16^ studied the frequency of ocular manifestations and the prognosis of secondary glaucoma in cases of CCF among patients from a multi-center group. Among their 43 patients diagnosed with CCF over a 16-year period, elevated IOP occurred in over 64% of patients having ocular involvement, which ranged from 22 to 55 mm Hg. Intraocular pressure control may be favorable in most of the eyes, with some exceptions, in which case, trabeculectomy may be necessary.

There may be venous and arterial stasis resulting in decreased ocular and retinal perfusion. Retinal and choroidal changes may include venous dilatation, retinal hemorrhage, central retinal vein occlusion, central retinal artery occlusion, cotton wool patches and serous retinal detachement.^1^,^5^,^19^,^20^ In addition there may be anterior segment ischemia due to decreased perfusion to intra-cavernous sinus cranial nerves, resulting in ophthalmoplegia and diplopia mimicking Graves' ophthalmopathy.^5^,^21^ The visual loss may be secondary to corneal, retinal or optic nerve changes or may result from the accompanying glaucoma.^3^–^21^ Development of CCF after cataract surgery may be uncommon. Nagaki *et al.*^22^ reported a case of CCF after cataract surgery in a 77-year-old woman who presented with acute proptosis and dilation of episcleral vessels that increased gradually. One month later, choroidal detachment developed in her eye. Computed tomography scan (CT scan) showed an enlarged SOV, and selective cerebral angiography showed fistulas between the meningeal branches of both the internal and external carotid arteries and the cavernous sinus. After the neurosurgical intervention, patient's symptoms resolved. Patients with CCF usually have more than one clinical sign or symptom. In a retrospective study, Preechawat *et al.*^13^ reported that almost all of their 80 patients with CCF had more than one clinical sign or symptom, and these included decreased vision in 43%, proptosis in 84%, arterialization of conjunctival vessels in 93%, chemosis in 42%, cranial nerve palsy in 52%, elevated IOP in 51% and optic neuropathy in 13% of the patients. The degree of visual deficit in their patients ranged from 20/40 to no light perception. Clinically silent cavernous sinus thrombosis can give rise to spontaneous indirect CCF.^11^ Patients with spontaneous indirect CCF may present with pulsatile tinnitus, temporal headache and ptosis. Imaging studies such as MRI angiography may be required in the proper diagnosis of these patients.

## CLASSIFICATION OF CCFs

Classification of CCFs is based on anatomical, hemodynamic and etiological factors.^1^–^5^ Anatomically they can be direct or indirect with high-flow or low-flow dynamics due to either traumatic or spontaneous causes. Barrow (1985) classified CCFs into 4 subtypes based on their communication. For example, type A CCF results from direct communication between ICA and cavernous sinus, type B results from a communication between dural ICA branches and cavernous sinus, type C results from communication between dural external carotid artery branch and cavernous sinus and type D results from a communication from dural branches of ICA and external carotid artery branches to cavernous sinus. In the direct CCF, arterial blood passes directly through a defect in the wall of intra-cavernous portion of the ICA. Because of CCF, blood in vein becomes arterialized, resulting in increased venous pressure, increased arterial pressure and decreased perfusion. Based on proper imaging studies, most CCFs can be classified into one of Barrow's classifications. In a study of consecutive patients having CCF, Preechawat *et al.*^13^ were able to classify their 80 patients according to Barrow's classification after angiographic evaluation, as type B- 14%, type C- 15% and type D- 71%.

## DIFFERENTIAL DIAGNOSIS OF CCF

Some of the differential diagnoses for CCF include vascular lesions such as arteriovenous malformation and cavernous sinus thrombosis, cavernous sinus tumors, orbital tumors, skull base tumors and mucocele.^5^,^21^ Mendicino *et al.*^3^ reviewed ocular manifestations of the most common intracranial vascular abnormalities: intracranial aneurysms, CCF, arteriovenous malformations and cavernous malformations. Direct and indirect CCFs most commonly cause the classic triad of proptosis, conjunctival chemosis and cranial bruit but can masquerade as chronic conjunctivitis (Figures 1–3). Unruptured aneurysms can compress the third cranial nerve and the anterior visual pathways. Ruptured aneurysms and subarachnoid hemorrhage can result in Terson syndrome and papilledema. Arteriovenous malformations, with or without hemorrhage, may compress portions of the retrochiasmal pathways, causing visual field loss. Cavernous malformations when in the brainstem commonly cause abnormalities of supranuclear, nuclear and fascicular ocular motility. Elderly patients may present with spontaneous arteriovenous fistulas of the orbit with very minimum symptoms. Ohtsuka and Hashimoto^5^ reported a case of a 73-year-old woman who had presented initially with a 1-year history of mild proptosis in the absence of any trauma. Careful ophthalmologic examination disclosed dilatation of conjunctival vessels, increased IOP, and bruit over the affected eye, which led them to the diagnosis of CCF. Imaging studies of this patient exhibited dilation of the SOV, and selective cerebral angiography disclosed communications between branches of both ophthalmic and facial arteries and the SOV in the orbit. Some of the traumatic causes may include retrobulbar hemorrhage and intra-orbital foreign body. Infections such as orbital cellulitis, mucormycosis and tuberculosis may also present as CCF. Thyroid eye disease; orbital pseudotumor; and orbital vasculitis resulting from Wegener's granulomatosis, polyarteritis nodosa, intracranial sarcoidosis and Tolosa-Hunt syndrome may present like CCF.^21^ Bhatti *et al.*^4^ reported a case of a 90-year-old woman with a 4-week history of a presumed infectious conjunctivitis resistant to topical antibiotic medications. Closer examination revealed tortuous, dilated conjunctival vessels; retinal hemorrhages; and an orbital bruit suggestive of a CCF. A cerebral arteriogram confirmed a direct CCF. They emphasized that their case illustrated the spectrum of subtle-to-conspicuous ocular manifestations that could be seen in patients with CCF and their potential to present as an emergency. Therefore, a CCF should be included in the differential diagnosis of an “atypical” red eye. Recognition of arterialized conjunctival vessels and auscultation of an orbital bruit raise the possibility of a CCF, requiring prompt diagnostic studies.

## IMAGING STUDIES

On imaging studies such as CT scan and MRI, CCF may present with enlarged SOV, thick extraocular muscles and evidence of enlarged cavernous sinus with a convexity of the lateral wall (Figures 2C and 2D 3C and 3D).^10^,^24^ These changes can only make one suspect a fistula. Serial dynamic enhanced CT (serial DE-CT) as a diagnostic tool for CCF has been found to be useful for the initial diagnosis of both high- and low-flow CCFs.^23^ MR angiogram may demonstrate some of the higher-flow fistulas but does not provide the detail necessary for complete evaluation and treatment (Figure 2G). Color Doppler images may demonstrate arterialized blood flow in dilated SOV and return of normal venous flow after successful treatment.^25^ Bilateral selective arteriography of both internal and external carotid arteries may be necessary to completely characterize the blood supply and drainage of a dural cavernous sinus arteriovenous fistula.^1^,^13^

## INDICATIONS FOR TREATMENT

Most CCFs are not life threatening, but the involved eye is at risk. Main indications for treatment include glaucoma, diplopia, intolerable bruit or headache, and severe proptosis causing exposure keratopathy. Spontaneous closure from thrombosis of cavernous sinus is unlikely (especially those that occur after trauma or in high flow fistulas). Dural fistulas may undergo spontaneous closure, especially after diagnostic angiography.^1^–^5^ Carotid compression therapy has also been successful in closure of 17% of direct and 30% of dural CCFs.^1^–^3^,^8^ Surgical treatment has included ligation of the external and internal carotid arteries; and fistula embolization with particles, glue, detachable balloons and thrombogenic microcoils.^1^,^8^,^25^–^27^ Direct fistulas are best treated with a detachable balloon through an endarterial route. This technique is successful in over 90% of cases, although there may be a transient ocular motor paresis in 30% of patients.^1^,^25^–^29^ Moron *et al*.^27^ reported their experience of using stent-assisted coil placement for treatment of 6 patients with high-flow CCFs that were associated with severe laceration of the ICA. They suggested that stent-assisted coil placement in these patients may offer a safe and effective treatment. Treatment of cavernous dural arteriovenous fistulas is usually done using a trans-arterial approach. However, in many complicated cases, treatments using trans-arterial approach may not be feasible, or are unsuccessful; but dural carotid cavernous fistulas can be treated with trans-venous embolization. He *et al*.^28^ studied the feasibility of embolizing 6 cases of complicated cavernous fistulas with a combination of detachable coils and onyx via a trans-venous approach. They were able to fully embolize 4 with onyx by a single operation, and 2 with onyx following two operations. Because onyx may be injected via a trans-venous approach and the micro-catheter is easily withdrawn, cavernous sinus embolization via trans-venous catheterization may be safe and efficient in treating complicated CCFs.

Retrograde cannulation of the SOV is an important route for embolization of cavernous sinus dural fistulas. Superior ophthalmic vein approach, first proposed by Hanneken *et al.* in 1989, enables a direct access to cavernous sinus and can be used to embolize by utilizing platinum microcoils.^27^ However, the procedure cannot be performed if the SOV is thrombosed; and there are a number of potential complications, which include difficulty in finding it, difficulty in determining the direction of the flow, orbital hemorrhage from inadvertent puncture of the SOV, orbital infection, possible injury to trochlea and loss of vision associated with acute orbital congestion secondary to thrombosis of the ophthalmic veins.^30^ Goldberg *et al.*^8^ reported a retrospective clinical series of 10 consecutive cases in which they were able to get access to the sinus through dilated SOVs by anterior orbitotomy with cannulation without any significant complications. Leibovitch *et al.*^7^ presented their clinical experience of technically difficult cases in which it was not possible to isolate or cannulate SOV for embolization. Among the 91 patients they studied, they were able to embolize only 25 via the SOV. In some circumstances, inferior ophthalmic vein may be used for catheterization if SOV is not accessible or thrombosed. Michels *et al.*^26^ accessed inferior ophthalmic vein via the inferonasal orbital space and were able to catheterize for delivery of multiple platinum coils to the cavernous sinus fistula of an elderly patient. Among the 80 consecutive patients reported by Preechawat *et al.,* clinical cure was achieved in 41 (51%) and improvement in 30 (38%) patients. Anatomical cure was demonstrated by angiogram in 50 (63%) patients.

Recently, Gralla *et al.*^29^ have reported on the use and efficiency of the Amplatzer vascular plugs in their series of 4 patients. Their experience suggested that these plugs may have potential for occlusion of large vessels and high-flow lesions in neuro-intervention. Navigation, positioning and detachment of the device were satisfactory in all of their cases. Sometimes, thrombosis of SOV or an anatomic variant may not allow its cannulation. Badilla *et al.*^31^ described a case of a cavernous dural fistula in which an anteriorly narrowed and thrombosed SOV was cannulated in the deep orbit through a lateral orbitotomy.

## COMPLICATIONS OF TREATMENT

Embolization of CCF may carry a risk of inherent complication either from the procedure or due to reopening of the fistula. Arruabarrena *et al.*^31^ reported a case of complete ophthalmoplegia and visual acuity loss due to a central retinal vein obstruction after an attempt to close a CCF. After a second embolization attempt, the fistula was closed successfully, but proptosis, chemosis and IOP remained uncontrolled despite medical treatment. Among the 80 patients treated by Preechawat *et al.,*^13^ intra-operative complications were found in 3 patients, which included ophthalmic artery occlusion and cerebral infarction. Eight patients experienced transient aggravation of symptoms, including increased proptosis, elevation of IOP, choroidal detachment that required suprachoroidal drainage, and venous stasis retinopathy. Ophthalmic vein thrombosis resulting in central retinal vein occlusion was developed in 3 patients and finally caused severe visual deficit. Although the SOV is a useful route for CCF embolization, the presence of fragile or clotted veins can preclude successful cannulation. Therefore, SOV embolization in the management of CCF may be associated with vision-threatening complications. Kikkawa *et al.*^32^ reported a case of a 69-year-old man who developed unilateral vision loss and neovascular glaucoma after attempted SOV embolization in the treatment of a CCF. Deeper orbital dissections carry a higher risk of uncontrolled bleeding and may need to be avoided, especially in older patients with fragile veins and those with recently diagnosed high-flow fistulas.

## ROLE OF AN OPHTHALMOLOGIST

Many patients with CCF may initially present to an ophthalmologist, who should be able to make a presumptive diagnosis in most cases. The ophthalmologist should be able to order appropriate tests to help make a diagnosis and characterize the features of the disease. In addition, the ophthalmologist should be able to monitor the course of the disease, including extraocular changes, fundus changes and IOP measurements. He should be able to treat glaucoma, which may be of several types. Although most cases are due to increased episcleral venous pressure, yet some are due to angle closure or iris neovascularization. In addition, the ophthalmologist should participate in the selection of patients that may be candidates for embolization treatment and possibly assist at surgery by helping isolate the ophthalmic vein at the time of surgery. When a patient is diagnosed with CCF, an ophthalmologist should properly refer the patient to a neurologist/neurosurgeon with assistance from a neuro-ophthalmologist. Based on patient's symptoms, the treatment may be observation, neuro-radiological intervention or neurosurgical intervention. These patients are followed up by ophthalmologists for persistence of any of their symptoms and continuous management if indicated. The management may involve treatment of glaucoma, exposure keratopathy and correction of persistent diplopia after resolution of CCF.
